# Intravenous Iodinated Contrast Induced Thyrotoxic Periodic Paralysis: A Case Report

**DOI:** 10.1155/2022/3615312

**Published:** 2022-10-03

**Authors:** S. Alrushaid, T. Alessa

**Affiliations:** ^1^Kuwait University, Kuwait City, Kuwait; ^2^Division of Endocrinology, Diabetes and Metabolism, Jaber Al-Ahmad Hospital, Kuwait City, Kuwait; ^3^Dasman Diabetes Institute, Kuwait City, Kuwait

## Abstract

Thyrotoxic periodic paralysis (TPP) is an entity that has been described in the literature as a transient, symmetrical, flaccid paralysis, mainly affecting the lower limbs of patients with a current or previous history of hyperthyroidism. In most cases, Graves' disease is the cause of hyperthyroidism. Contrast and iodine-induced TPP have been described in the literature, but only one case of intravenous contrast induced TPP has been reported. We report a case of TPP following administration of intravenous contrast for a computed tomography scan of the neck prior to lymph node excision. A 35-year-old Kuwaiti male with known Graves' disease in remission until two months of his presentation, reported to the emergency room one early morning in December 2020. He sustained a fall from the stairs due to bilateral lower limb weakness, mostly proximal. The upper limbs were spared, and the patient did not experience any numbness or headache. His potassium was found to be 2.1 mmol/L and an electrocardiogram showed U waves and ST segment changes. He was initiated on 20 mEq of intravenous potassium chloride in 500 mL sodium chloride over one hour, following which his potassium approached normal and his weakness resolved. He was last known to be euthyroid in November 2019 but noted in October 2020 to be in the hyperthyroid state when thyroid function testing showed a thyroid-stimulating hormone of <0.005 (0.27–4.2 uIu/mL) and free thyroxine (T4) of 27.6 (7.8– pmol/L). In patients with known hyperthyroidism, more caution is required when iodine-containing substances are administered without proper evaluation of thyroid function.

## 1. Case Report

A 35-year-old Kuwaiti man with known Graves' disease, brought by ambulance to the emergency room (ER) in Mubarak Al-Kabeer Hospital early morning in December 2020 with an acute episode of bilateral lower limb weakness. The weakness was of a sudden onset, first noticed as the patient was getting up from a seated position late at night. On walking, the patient noticed his legs felt heavy. Upon attempting to go up the stairs, his legs were weak and gave away, causing him to fall and sustain some minor injuries. There was no associated numbness or paresthesia, blurry vision, or speech disturbances. His upper limbs were completely free from any symptoms. He denied any symptoms of hyperthyroidism.

Before the event, he denied consuming a high carbohydrate meal, but mentioned drinking a small cup of milk with tea 2–4 hours before his symptoms began. He also denied exercise or alcohol consumption prior to the incident. However, he did receive 80 mL of low osmolality iodinated intravenous (IV) contrast (Visipaque) in the morning of the incident for contrast computed tomography (CT) imaging of the neck at Al-Sabah Hospital, as a surgery for excising an enlarged isolated cervical lymph node was planned. Visipaque is available in concentrations of 270 and 320 mg of organically bound iodine per mL (550 and 652 mg of iodixanol per mL, respectively). No discussion of his hyperthyroidism condition was performed by the radiology department before the CT procedure.

In the ER, his temperature was 36.8 degrees C, heart rate 86 bpm, and oxygen saturation 99%. The initial basic blood chemistry test was done showing low potassium (2.1 mmol/L). Electrocardiogram (ECG) was performed twice, initially showing *U* waves and later ST segment abnormalities.

Two months prior in October 2020, the patient was admitted to a local hospital for the diagnosis of acute gastroenteritis. He presented with symptoms of abdominal pain, vomiting, and diarrhea. He was managed conservatively with intravenous fluids. During admission, several laboratory tests were done, including a thyroid function test showing thyroid-stimulating hormone (TSH) < 0.005 (0.27–4.2 uIu/mL) and free thyroxine (FT4) 27.6 (7.8–16 pmol/L). The caring medical team decided not to use antithyroid medications as it was believed to be a transient hyperthyroidism related to his acute illness.

The patient was diagnosed with hyperthyroidism due to Graves' disease in 2016 after presenting with palpitations, sweating, anxiety, and agitation. At the time, his TSH was 0.12 (0.27–4.2 uIu/mL), FT4 of 14.1 (7.8–16 pmol/L), and free T3 of 8.0 (3.5–6.7 pmol/L). Thyroid uptake scan showed homogenously increased radiotracer distribution in both lobes, with no discrete areas showing decreased or increased uptake. Four-hour and 24-hour radio-iodine uptakes were 33.6% and 62%, respectively. These findings were consistent with a diffuse, toxic thyroid gland, diagnosed as Graves' disease. Medical therapy with carbimazole was initiated. He was maintained euthyroid and kept on medical therapy. The patient and his caring endocrinologist have not planned an alternative therapy for the management of his hyperthyroidism. In 2018, thyroid ultrasound showed normal sized thyroid lobes but coarse echotexture and geographic hypoechoic areas with increased vascularity, suggesting thyroiditis. He was taken off carbimazole therapy in early 2019, and his thyroid function test was within the reference range since then. During the patient's last visit to his endocrinologist in November 2019, he did not report any symptoms of hyperthyroidism nor ophthalmic symptoms. His thyroid function test done during that visit showed a TSH of 0.95 (0.27–4.2 uIu/mL) and free T4 of 11.6 (7.8–16 pmol/L). His thyroid gland was mildly diffusely enlarged, nontender with no audible bruit or nodularity.

The patient also has hypertension and hyperlipidemia, both reported to be controlled on medical therapy with telmisartan 40 mg once daily and rosuvastatin 20 mg once daily. He does not use any illicit drugs or consume alcohol. He smokes an electric cigarette for two years. The patient had no previous surgeries except for laser-assisted in situ keratomileusis (LASIK) for a refractive error of his both eyes. He is married with two children and works at the Ministry of Education.

Both patient's parents have hypothyroidism diagnosed during adulthood, with no history of thyroid surgery. His mother had nephrectomy for a malignant renal tumor. There is no reported family history of other renal conditions or potassium abnormalities. His father is 74 years old and became bed-ridden after a stroke. The patient reported no family history of similar presentation of temporary paralysis.

## 2. Investigations

Initial basic blood chemistry performed in the ER (Table 1) was significant for hypokalemia (2.3 mmol/L). Elevated random serum glucose (8.9 mmol/L) and low phosphate (0.73 mmol/L) were also noted. The anion gap was elevated (20), although serum bicarbonate was normal (26 mmol/L). A few hours later, laboratory results showed elevated magnesium (1.37 mmol/L), low albumin (34 g/L), and normal corrected serum calcium (2.27 mmol/L). Electrocardiography (ECG) showed *U* waves and ST segment abnormalities.

## 3. Outcome and Follow-Up

In the ER, the patient was administered 20 mEq potassium chloride (KCl) in 500 mL sodium chloride (NaCl) intravenously over one hour and 2 g magnesium sulfate (MgSO4) over half an hour soon after the initial test result, which allowed gradual improvement of his weakness until resolution with improvement of his serum potassium (Table 1) in less than four hours. Serum magnesium levels noted to be elevated (1.37 mmol/L) possibly due to MgSO4 infusion but no baseline level was available. Patient was connected to continuous cardiac monitoring during treatment of hypokalemia and no cardiac arrythmias or abnormal findings were detected during the potassium replacement therapy. Beta-blocker were not administered during his ER stay. The patient was discharged home from the ER on paracetamol and advised to follow a high potassium diet. No potassium supplements were given. He did, however, sustain superficial bruises with pain in his lower extremities that lasted for 48 hours afterwards.

Two weeks following the event, he was evaluated by another endocrinologist, and a new thyroid function test performed showing TSH <0.005 (0.27–4.2 uIu/mL) and FT4 of 13 (7.8–16 /L) indicating most likely a resolving hyperthyroidism. He was not started on any antithyroid therapy. His TSH values improved over the next eight months reaching 1.32 (0.27–4.2 uIu/mL) with normal FT4 in August 2021.

## 4. Discussion

Thyrotoxic periodic paralysis (TPP) is an entity that has been described in the literature as a transient, symmetrical, flaccid paralysis, mainly affecting the lower limbs of patients with a current or previous history of hyperthyroidism [1, 2]. In most cases, Graves' disease is the cause of hyperthyroidism [3]. Other causes include toxic multinodular goiter, thyroiditis, or iodine-induced thyrotoxicosis [4]. It is more common in people of Asian descent and following a carbohydrate-rich meal, which results in an increase in the insulin level [5, 6]. Patients tend to present during early morning hours, as a catecholamine peak occurs during this time [3]. Both insulin and catecholamines are known to inhibit the Kir channel in the skeletal muscle, a channel that allows potassium to move extracellularly, which could explain why a rise in either could precipitate hypokalemia in susceptible people [3]. The cardinal finding is hypokalemia and the culprit in this case is thought to be a rise in thyroid hormone levels [7, 8]. The severity of the weakness correlates with the degree of hypokalemia [5, 7]. During thyrotoxicosis, there is an increase in transcription of Na/K ATPase pumps as well as increased activity through two mechanisms: directly by thyroid hormones and via the resulting increased B2-adrenergic receptor sensitivity [4, 7, 9]. The influx of potassium into the intracellular compartment results lowered the levels of plasma potassium, leading to periodic paralysis [1]. The increased Na/K ATPase activity allows for more intracellular displacement of potassium, causing lower plasma potassium levels which leads to periodic paralysis [1, 3, 4, 7]. Genetic changes have been identified in TPP, involving the Na/K ATPase pump and Kir channel [3, 4, 7]. The Kir channel normally allows potassium to flow out of the cell, so loss of function mutations involving the Kir channel gene predispose to TPP [3, 4, 7]. This condition has a male predominance which makes these genetic alterations possibly X-linked, or that sex hormones play a role in the pathophysiology. In fact, androgens are known to increase the activity of the Na/K ATPase pump, while estrogen and progesterone inhibit it [3, 9, 10]. Furthermore, androgens induce muscle growth and hypertrophy, which increases the amount of Na/K ATPase pumps available for stimulation [10].

TPP is often confused with familial periodic paralysis (FPP) [11]. FPP occurs due to autosomal dominantly inherited mutations in skeletal muscle sodium, potassium, or calcium receptors [11, 12]. It may be hypokalemic, hyperkalemic, or normokalemic [11, 12]. The associated transient paralysis lasts anywhere from minutes to days [12]. Family history of thyroid conditions, rather than of transient paralysis may be helpful in distinguishing the two entities, especially since familial periodic paralysis has an autosomal dominant inheritance pattern [1, 7, 11]. History or findings of hyperthyroidism is also useful [7, 11]. Both patient's parents have hypothyroidism, both had it as a primary condition and not proceeded by hyperthyroidism. No reported transient paralysis was noted in the patient's family history. In addition, hypomagnesemia and hypophosphatemia tend to occur in TPP only due to intracellular shift from a catecholamine increase [4, 5]. The initial laboratory findings for our patient did not include magnesium test but his phosphate was low. Patients with TPP also tend to have a preserved acid-base balance as well as low creatinine [5, 7]. In our patient, the anion gap was elevated while the bicarbonate level was normal. This observation could not be explained due to lack of further clinical and laboratory data.

During the attack, patients may present with signs and symptoms of hyperthyroidism, such as palpitations, tremors, and tachycardia [11, 13]. Our patient, however, did not experience any symptoms of hyperthyroidism during the attack apart from systolic hypertension in the emergency room, which made the diagnosis challenging. Most patients with TPP have only mild or subclinical hyperthyroidism but our patient had a significant hyperthyroidism thyroid profile with suppressed TSH and FT4 double the upper normal range detected two months before his presentation [5, 14]. This could have facilitated the development of TPP in our patient. With correction of the hypokalemia, affected patients classically regain complete motor function in their lower limbs within hours, sometimes up to days [3].

There are multiple reported cases of iodine-induced TPP after ingestion of amiodarone and other iodine-containing substances, such as radioactive iodine and kelp [1, 14–18]. However, only one case of TPP following administration of intravenous iodine-containing contrast has been reported which was also managed urgently with potassium replacement [19]. In that case, proximal weakness of both his upper and lower limbs with inability to stand independently one day after cardiac catheterization with iodinated contrast was reported [19]. Our patient's presentation spared the upper limbs. Other reports of TPP mentioned using beta-blockers, which was not used in our Case [1]. Beta-blockers are given to cause intracellular shifting of potassium and counteract the increased B-adrenergic receptor sensitivity [1].

The Jod–Basedow phenomenon can be used to explain the pathophysiology of iodine-induced TPP [20]. Normally, those exposed to large amounts of exogenous iodine will experience a transient reduction in thyroid hormone synthesis [20]. This inhibition is independent of TSH and is called the Wolff–Chaikoff effect [20]. Patients with hyperthyroidism could escape this effect as thyroid hormone synthesis is not controlled by pituitary TSH, and comparatively large quantities of iodine or iodide when ingested can lead to iodine-induced hyperthyroidism (Jod–Basedow effect) [19]. Our patient had significant hyperthyroidism before receiving the intravenous iodine contrast, escaping the Wolff–Chaikoff effect would be likely and optimal precontrast preparation was required.

Intravenous potassium replacement is the main element in the management of TPP and needs to be done as soon as possible; however, as these patients' total body potassium is normal, rebound hyperkalemia may result from potassium replacement along with eventual extracellular shifting [3]. Inhibitors of thyroid hormone synthesis are given to treat the underlying cause [3]. It is unclear whether potassium supplementation is helpful prophylactically; however, beta-blockers and antithyroid medication are beneficial in the long-term [3, 10, 19]. Our patient was only advised to maintain a potassium-rich diet after the episode. Furthermore, in those with contrast or iodine-induced TPP, this should be noted for more precautions to be taken in the future. Our patient showed laboratory features of improving hyperthyroidism in the months following the event, it is not clear whether his relapse was transient and remitted spontaneously.

TPP is a challenging diagnosis, especially in the emergency setting. Intravenous contrast-induced TPP is even more challenging. Increasing the awareness of patients and their physicians is crucial in allowing timely diagnosis and management. As most patients will not have overt hyperthyroidism, this must be sought out using a high index of suspicion based on previous history of thyroid disease in a patient with acute weakness or paralysis. Proper investigations to differentiate between thyrotoxic and familial forms of periodic paralysis are helpful and should be carried out. As the paralysis is transient, patients must be reassured and educated following the episode. In patients with documented hyperthyroidism, more caution is required when iodine-containing substances are administered without proper control of thyroid function.

## Figures and Tables

**Table 1:** Serum laboratory findings in the emergency room.

Tests	6 : 48 AM	7 : 25 AM	10 : 16 AM	11 : 05 AM	Reference range
Glucose		8.9			4.1–5.9 mmol/L
Urea		4.8			2.8–7.2 mmol/L
Creatinine		84			64–104 *µ*mol/L
Sodium		141			136–146 mmol/L
Potassium	2.10	2.31	3.50		3.5–5.1 mmol/L
Chloride		104			101–109 mmol/L
Phosphate		0.73			0.81–1.45 mmol/L
Calcium				2.15	2.2–2.65 mmol/L
Magnesium				1.37	0.73–1.06 mmol/L
Corrected calcium				2.27	2.2–2.6 mmol/L
Albumin				34	35–52 g/L
CO2		28			21–31 mmol/L
Anion gap		11.3			

## Data Availability

The medical data used to support the findings of this case study are included within the article.
